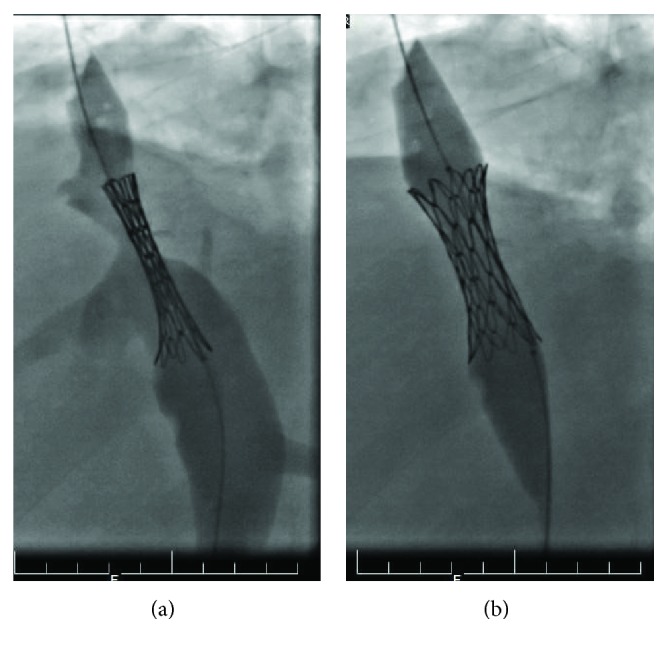# Intravascular Thrombolysis Followed by Stenting as Management of Retrohepatic Inferior Vena Caval Thrombosis due to a Twist in the Inferior Vena Cava after Deceased Donor Liver Transplant

**DOI:** 10.1155/2019/7292974

**Published:** 2019-06-17

**Authors:** Olivia Kituuka, Rakesh Rai, Ashok Thorat, Vianney Kweyamba, Alex Elobu, Prabath Mondel

**Affiliations:** ^1^Department of Liver Transplantation, Fortis Hospital, Mulund-Goregaon Link Road, Mulund West, 400080 Mumbai, India; ^2^Fortis Hospital Mulund, Mumbai, India

## Abstract

Inferior vena cava (IVC) occlusion due to acute thrombosis is a rare but important vascular complication after deceased donor liver transplantation (DDLT) that has been reported to occur up to 2% of recipients in a posttransplant period. This may be caused by direct instrumentation of the IVC stenosis at the anastomotic site, haematoma, and rarely by a twist in the retrohepatic IVC. The location of the thrombus, the timing after the surgery, and associated hemodynamic disturbances define the outcome of the patient. Without prompt diagnosis and timely intervention, the outcome after IVC thrombosis is usually dismal. Herein, we report a rare case of near-complete occlusion of the IVC secondary to intracaval thrombosis after DDLT associated with twisting of the IVC at the suprahepatic anastomosis which was successfully managed by intravascular thrombolysis and stenting.

## 1. Introduction

### 1.1. Case Report

**Figure 1:**
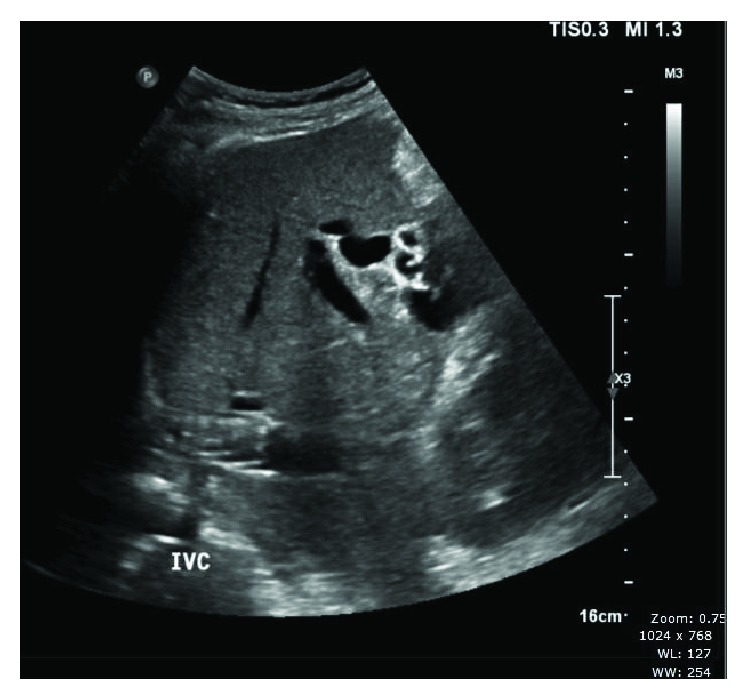


A 59-year-old male underwent uneventful DDLT for decompensated hepatitis B-related liver cirrhosis with a MELD score of 19. The transplant surgery and postoperative course were uneventful. He was discharged on the 11^th^ posttransplantation day with stable liver function. Immunosuppressive protocol was as per standard institution protocol. The patient was admitted after 6 weeks in an emergency department with hypotension, vomiting, and altered sensorium associated with oliguria. Liver functions were grossly elevated, and he had a systolic hypotension of 70 mmHg. Emergency abdominal ultrasound scan showed mild hepatomegaly and an echogenic thrombus in the retrohepatic IVC near the suprahepatic anastomosis (Figure 1) which was confirmed by computed tomography (CT) angiography that also revealed renal vein and iliac vein thrombosis as an incidental finding.

**Figure 2:**
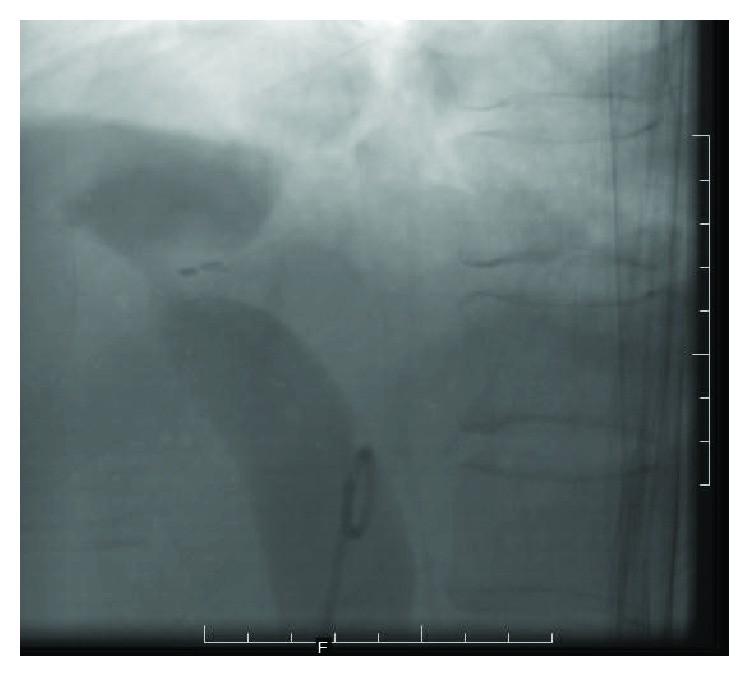


The patient was admitted in ICU and started on anticoagulation therapy, and a digital subtraction inferior venacavogram was done. This showed a focal severe stenosis approximately 70% in the inferior vena cava at the level of the T12 vertebra. There was an associated thrombus 6 × 3 cm within the retrohepatic and suprahepatic inferior vena cava with complete cut-off of the inferior vena cava 3 cm proximal to its junction with the right atrium. Intravascular thrombolysis using urokinase 50,000 IU was immediately instituted, and the patient was maintained on 100,000 IU/hour of urokinase infusion in the IVC with repeated mechanical thromboaspiration. The patient remained stable during the thrombolysis, and there was reestablishment of blood flow across the previously occluded part of the IVC. Post procedure abdominal ultrasound and Doppler ultrasound were done after 48 hrs, and both still showed the presence of a thrombus. Abdominal CT scans also showed severe stenosis in the suprahepatic inferior vena cava just proximal to the right atrial junction with mild to moderate ascites and splenomegaly. The patient had another angiography done which confirmed a 2 cm short segment severe stenosis of about 95% in the IVC at its junction with the right atrium (Figure 2). The IVC was twisted along its long axis in this region.

He then underwent inferior vena cava venoplasty, and an endovascular stent was inserted whereby a balloon-mounted stent was deployed across the stenosis site to a size of 18 × 32 mmm (Figure 3(a)). Post stenting, there was improvement in the calibre of the IVC and it was untwisted (Figure 3(b)). There was no pressure gradient proximal and distal to the stenosis.

Following the procedure, the patient was given low molecular weight heparin 2500 IU subcutaneously for 3 weeks. The patient made uneventful recovery and was discharged 2 weeks post stenting on warfarin with a target international normalized ratio between 2 and 2.5. The liver functions were normal at the time of discharge.

Following the procedure, the patient was given low molecular weight heparin 2500 IU subcutaneously for 3 weeks. The patient made uneventful recovery and was discharged 2 days post stenting on warfarin with a target international normalized ratio between 2 and 2.5. The liver functions were normal at the time of discharge.

Six weeks after discharge, he was reviewed and had no complaints. An abdominal ultrasound done showed no thrombus in the inferior vena cava and the rest of the abdominal organs were normal as well as his liver and renal function tests.

## 2. Discussion

Inferior vena cava thrombosis is a rare but potentially fatal complication of liver transplant occurring in up to 2% of liver transplant patients. It may be caused by instrumentation of the IVC, haematoma formation, or stenosis of the retrohepatic IVC [1, 2]. Stenosis associated with twisting of the IVC is a rare technical complication which can occur at the site of the anastomosis especially in the suprahepatic anastomosis as described in this patient [1, 2]. There are a few case reports about twisting or stenosis being the cause of IVC thrombosis with the commonest site being the retrohepatic IVC [3]. In this patient, the stenosis and twist and the resultant thrombus occurred at the junction of the IVC with the right atrium. Unlike most cases of right atrial thrombus which occur intraoperatively during liver transplantation, only one case has been reported of a thrombus occurring at this site over a month post liver transplant. As in our patient, the thrombus in the right atrium arose from the IVC [4]. Patients may present as asymptomatic with incidental finding of the thrombus or may get dizziness, ascites, and hepatic dysfunction which may be acute or chronic if the compression of the IVC is gradual [1, 5]. Doppler ultrasound is the modality of choice for identifying vascular complications post liver transplantation and will identify kinking and thrombus in the liver vasculature [6]. In this case, an emergency ultrasound with Doppler of the liver was able to identify the thrombus in the IVC. Percutaneous vascular stenting of IVC stenosis after liver transplantation as a method for correcting the signs and symptoms of IVC thrombosis has been shown to be efficacious and has also demonstrated long-term patency of the IVC post stenting [5, 7, 8]. Stenting is a safe and effective way of treating torsion, compression, and stenosis of the IVC following liver transplant [9].

## 3. Conclusion

Twisting of the retrohepatic IVC can occur after DDLT and predisposes one to acute thrombosis that may prove fatal. Intravascular thrombolysis using urokinase followed by stenting is the feasible treatment modality in such patients and also relieves the torsion.

## Figures and Tables

**Figure 3:**